# Assessing the Impact of Movement Consequences on the Development of Early Reaching in Infancy

**DOI:** 10.3389/fpsyg.2016.00587

**Published:** 2016-04-27

**Authors:** Joshua L. Williams, Daniela Corbetta

**Affiliations:** ^1^Department of Psychology, Armstrong State UniversitySavannah, GA, USA; ^2^Department of Psychology, The University of TennesseeKnoxville, TN, USA

**Keywords:** motor development, infants, reaching, reinforcement learning, contingent reinforcement, developmental trajectories, sensorimotor experience

## Abstract

Prior research on infant reaching has shown that providing infants with repeated opportunities to reach for objects aids the emergence and progression of reaching behavior. This study investigated the effect of movement consequences on the process of learning to reach in pre-reaching infants. Thirty-five infants aged 2.9 months at the onset of the study were randomly assigned to 1 of 3 groups. Two groups received a 14-day intervention to distinct reaching tasks: (1) in a contingent group, a toy target moved and sounded upon contact only, and (2) in a continuous group, the toy moved and sounded continuously, independent of hand-toy contact. A third control group did not receive any intervention; this group’s performance was assessed only on 2 days at a 15-day interval. Results revealed that infants in the contingent group made the most progress over time compared to the two other groups. Infants in this group made significantly more overall contacts with the sounding/moving toy, and they increased their rate of visually attended target contacts relative to non-visually attended target contacts compared to the continuous and control groups. Infants in the continuous group did not differ from the control group on the number of hand-toy contacts nor did they show a change in visually attended target versus non-visually attended target contacts ratio over time. However, they did show an increase in movement speed, presumably in an attempt to attain the moving toy. These findings highlight the importance of contingent movement consequences as a critical reinforcer for the selection of action and motor learning in early development. Through repeated opportunities to explore movement consequences, infants discover and select movements that are most successful to the task-at-hand. This study further demonstrates that distinct sensory-motor experiences can have a significant impact on developmental trajectories and can influence the skills young infants will discover through their interactions with their surroundings.

## Introduction

In typically developing infants, reaching emerges between 3 and 5 months of age (von Hofsten, 1984; Clifton et al., 1993; Thelen et al., 1993). The appearance of this behavior has significant cascadic effects on many areas of development. For example, it impacts gains in motor control and the emergence of novel exploratory abilities (von Hofsten, 1991; Konczak et al., 1995; Thelen et al., 1996; Bhat et al., 2005), it offers new opportunities to learn about object properties (Gibson, 1988; Rochat, 1989; Bushnell and Boudreau, 1993; Lederman and Klatzky, 1993; Corbetta and Snapp-Childs, 2009; Lobo and Galloway, 2013), and triggers changes in the socioemotional context (Bakeman and Adamson, 1984; Fogel et al., 1992; Ruff and Rothbart, 1996; Fogel, 1997). Because of the conspicuous importance of such a behavior, developmental scientists have actively sought to understand how it forms in infancy. For many decades, researchers have carefully described the progression of this behavior over time by focusing mainly on the role that vision played in the guidance of the arm toward the target object (e.g., Piaget, 1952; White et al., 1964; von Hofsten, 1979, 1982, 1984; Bushnell, 1985). Since the late 1990s, however, researchers have come to understand that the emergence of reaching is the product of multiple interacting subsystems, not just vision (Thelen, 1992, 1995; Thelen and Smith, 1994, 1998; Spencer et al., 2000; Clearfield and Thelen, 2001; Cunha et al., 2015). As a result, research efforts have increasingly shifted toward identifying which types of early experiences can help the integration of these multiple systems in fostering the emergence of infant reaching.

Several groups of researchers have now published studies that examined the impact of varied types of sensory-motor experiences on the emergence of infant reaching. One such study from Lobo et al. (2004) discovered that both general movement and reaching-specific, or object-directed, movement experiences served to drive higher amounts of reaching behavior relative to a no experience control. However, infants in the reaching-specific condition, in which the successful movement consequence was hand-toy contact, displayed significantly higher amounts of reaching relative to infants in the general movement experience condition. Furthermore, Lobo and Galloway (2008) replicated the reaching-specific results of Lobo et al. (2004) but extended the findings to show that infants who received specific reaching experience, and even basic postural experience, significantly outperformed infants who received non-object-directed social experience.

These studies revealed that broad, non-specific arm movements and postural enhancements – two contributing sub-systems to learning to reach – can help the emergence and development of reaching, but specific reaching experiences always led to faster learning outcomes. Along the same line of inquiry, another group of studies also found that reaching-specific experience interventions significantly helped precipitate the development of reaching (Cunha et al., 2013, 2015; Soares Dde et al., 2013). More unexpectedly, these studies discovered that even very short-term durations of 1 or 2 days of reaching-specific intervention sufficed to drive significant results in the amount of reaching performed in babies, compared to control babies who only received a non-object-directed social intervention.

The fact that reaching-specific interventions seem to have an impact on learning to reach fits well with the idea that repeated opportunities to reach for and explore specific action consequences may facilitate the discovery of successful actions (Schlesinger et al., 2000; Bojczyk and Corbetta, 2004; Corbetta and Snapp-Childs, 2009; Williams et al., 2015a). Needham et al. (2002) and Libertus and Needham (2010, 2014) further examined this idea through a series of studies where they fitted pre-reaching infants with “sticky mittens” thereby providing *simulated grasping* experience when the object stuck to the mittens following hand-toy contact. In these studies, the action consequence during the learning to reach process was not just limited to making hand-toy contact, but also offered infants the enhanced ability to seemingly *pick up* the toy. Parents were instructed to provide 10 min of reaching exposure to their infant wearing the “sticky mittens” for 12–14 consecutive days. The “sticky mittens” group was also compared to other age-matched groups of infants who received other kinds of “more passive” experiences. In one study (Libertus and Needham, 2010), the object was placed by the parent directly in the infant’s hand while wearing the mittens. In another (Libertus and Needham, 2014), the object was attached to the wrist of the infant, or in another condition, the infant was not encouraged to reach. In all study variations, performances were always compared with an age-matched, no intervention control group. The researchers consistently found that infants in the “sticky mittens” group performed more toy-directed behaviors than infants in any of the other groups, which led them to conclude that the simulation of grasping provided by the mittens served to drive increased toy-directed behavior.

In these studies, however, it remained unclear if the “sticky-mittens” experience provided something truly additional to the learning to reach experience. Much of the other group interventions to which the “sticky mittens” were compared did not entail much reaching behavior. Further, many aforementioned studies reported increases in learning to reach simply by exposing infants to classic, reaching-specific experiences. If we follow the reasoning that the consequences of an action are an important factor in driving the learning to reach process, then one may ask what could be the relative impact of the “sticky mittens” simulated grasping experience on the formation of initial reaching behavior, compared to simply touching the target. In an effort to address this question, Williams et al. (2015b) examined the developmental trajectories of near-reaching infants receiving task-specific reaching experience wearing “sticky mittens” with an age-matched group of infants who wore “non-sticky mittens.” Both groups received 14 days of 10-min, experimenter-led exposure to the reaching task. In addition, Williams et al. (2015b) recorded the arm movement kinematics prior to and after the 14-day reaching experience. These researchers found that both mittens groups displayed significant gains in the amount of visually attended target reaching over the course of the study, however, only infants in the “non-sticky mittens” group showed a significantly higher amount of visually attended target reaching relative to the no-experience control group on the final day. The “sticky mittens” group did not. In addition, infants in the “non-sticky” group showed a decrease in movement speed between the first and last day of the study, as did the no-experience group, which is an indication of improved movement control. Infants in the “sticky” group, on the contrary, increased movement velocity between the first and last day, suggesting that they were possibly learning to swipe more at the toy to pick it up rather than slowing movement speed to contact the toy accurately (Williams et al., 2015b).

These results indicated again that varying experiences associated with distinct movement consequences of hand-toy contact could drive diverse developmental trajectories in the early learning to reach process. Specifically, Williams et al.’s (2015b) study revealed that learning to reach was not particularly enhanced by the provision of grasping simulation, but that making direct contact with the toy alone was sufficient to drive the process of action selection. Further investigations of the “sticky” group’s performance led these researchers to pin point more accurately what might have driven the observed differences between mitten groups (Corbetta et al., 2015). Williams et al. (2015b) designed their mittens differently than the Needham group; the Williams et al. (2015b) mittens had openings for the fingers allowing infants in both groups to make direct haptic contact with the target depending on how the hand was directed at the toy at contact. Follow-up analyses revealed that the best performers in the “sticky mittens” group were the infants who made more direct bare finger contacts with the toy relative to simulated grasps. The data also revealed that the grasping simulation intervention with the open fingers mittens worked successfully – the toy stuck to the mitten at contact – but success at “picking-up” the toy via “sticky-mittens” with rare direct fingers-to-toy contact did not contribute to increased performance over time. Thus, those analyses indicated that reaching progression was driven more by direct haptic finger contact with the toys than by the provision of grasping simulation via “sticky-mittens” (Corbetta et al., 2015). This finding was in line with Schlesinger and Parisi’s (2001) work indicating that tactile feedback is an important factor in driving the exploration and selection of reaching movements. Through this series of studies we learned that infants may indeed rely on the consequences of their actions to increasingly select their actions, but these consequences may be more directly tied to direct haptic hand-toy contact than grasping simulation *per se*. This finding is in line with the findings of the aforementioned groups of researchers who observed progression in reaching-specific interventions without “sticky mittens” (Lobo et al., 2004; Lobo and Galloway, 2008; Cunha et al., 2013, 2015; Soares Dde et al., 2013).

This line of research has theoretical implications. We know that the process through which novel behavior emerges and organizes is complex and that it begins in early development through repeated cycles of action and perception, during which infants learn about their actions and their associated consequences (Gibson, 1988; Gibson and Pick, 2000; Corbetta, 2009). When infants discover action consequences relevant to the task-at-hand, those actions become selected over time and used in future, similar situations. Dynamic Systems Theory, for example, purports that the selection process leading to more sophisticated levels of reaching behavior is heavily driven by repeated cycles of action and perception (Bojczyk and Corbetta, 2004; Corbetta and Snapp-Childs, 2009). Such repeated cycles are also tied to the process by which the brain learns, and the values it attributes to the consequences of actions. Recent neuroscientific research, specifically, perspectives on neural substrates of behavioral development such as Edelman’s (1987) Theory of Neuronal Group Selection (TNGS) and Approximate Optimal Control Theory (Berthier et al., 2005) supplement Dynamic Systems to better explain the early emergence and development of behavior (see Williams et al., 2015a, for a more detailed account). In effect, both TNGS and Approximate Optimal Control provide potential neural mechanisms for the neuronal selection process that underlies behavioral change. Specifically, Edelman (1987) proposed that synaptic connections active during a successful behavior will be strengthened through signals sent from innate value systems which indicate that the most recent behavior performed was functionally valuable. Thus, those connections that receive signals from the positively activated value systems will be strengthened and more likely to be re-activated in similar future situations (Edelman, 1987; Sporns and Edelman, 1993). Approximate Optimal Control perspectives contribute to this view by providing a more continuous look at the selective process by applying principles of reinforcement learning to behavioral modeling technology. As a behavior aimed at a target is performed, a value function is created via continuous neural mapping of each system state during the behavioral sequence in relation to the goal. Behaviors during periods of activity that bring the infant closer to goal attainment are assigned a higher value and thus, the selection process is continuous and proceeds based on the associated potential reward returned by the value function based on the current state of the system (Berthier et al., 2005).

Many findings in other areas of motor development are consistent with such theoretical views. For example, research on infant kicking and early eye-hand coordination has shown that from very early in life, infants are able to engage in exploratory actions, discover the consequences of their actions, and select those actions that are adaptive to the task-at-hand (Rovee and Rovee, 1969; Thelen, 1994; van der Meer et al., 1995; Angulo-Kinzler, 2001). These studies clearly highlighted the importance of exploratory opportunities to the discovery and emergence of new skills in novel tasks. Discovery of new actions or patterns of action occurred through the exploration of varied movements and their consequences.

Bojczyk and Corbetta (2004) exemplified the importance of opportunities to discover movement consequences when they examined the impact of minimal, but repeated opportunities to explore an object-retrieval task on the emergence of successful bimanual coordination retrieval strategies. Prior research indicated that infants did not display such well-coordinated bimanual strategies in object-retrieval tasks until they reached 12–18 months of age (Bruner, 1970; Diamond, 1991). Bojczyk and Corbetta (2004) provided infants, beginning at 6 ½ months of age, with only six trials of weekly exposure to an object-retrieval task requiring bimanual coordination to retrieve a toy concealed in a box. They followed infants until they were able to perform well-coordinated bimanual patterns consistently. Compared to age-matched control groups that did not receive repeated exposure to the object-retrieval task, infants with repeated exposure showed significantly more well-coordinated bimanual strategies and they displayed these efficient strategies by the age of 8 and 9 months, which was much earlier than the ages of success reported for similar behaviors by prior object-retrieval studies (Bruner, 1970; Diamond, 1991). Thus again, repeated exposure to the task, which provided opportunities for the infants to perform various actions during the object-retrieval task, seemed to be enough to aid the selection process and enhance the development of successful bimanual coordination in much younger infants. In other words, through repeated actions and perception of action consequences, infants developed a value function that became tailored to the object-retrieval task and, in turn, facilitated the discovery of the most adaptive retrieval strategies for the task-at-hand. Gradually, over time the more successful strategies became increasingly selected and used more frequently (Bojczyk and Corbetta, 2004).

In the current study, we aimed to further explore the notion that direct hand-toy contact provides value for driving movement exploration, discovery, and selection of adaptive reaching responses around the time of the emergence of reaching. Specifically, we hypothesized that if hand-toy contact is particularly important for learning to reach, then emphasizing the consequence of such direct hand-toy contact may help precipitate the selection process and trigger a steeper developmental curve in reaching. If the engine of the selective process is the repetition of action and perception cycles in relation to discovered valuable action consequences, then task manipulations designed to highlight different movement consequences should spark and drive action-perception cycles selecting distinctive movement processes. With this scope in mind, this study aimed to manipulate the consequences of the immediate hand-toy contact to assess how variations in such movement consequences would lead to distinct developmental outcomes or different kinds of movement enhancement. We rooted our sensory-motor manipulations in two well-established lines of empirical research in order to examine the impact that each enhancement would have on the early reaching selection process.

First, work in the mastery motivation literature revealed that exposure to responsive toys, or toys activated contingently upon infants’ actions, in the everyday environment drove higher levels of task persistence during the first year of life (Jennings et al., 1979). In this work, the researchers operationalized *persistence* as the continued search for feedback from objects. Thus, in the context of early reaching, exposure to responsive toys at contact may increase infants’ persistence at reaching for and activating the toys and consequently enhance the discovery and selection process. To examine the initial trajectory of reaching as a function of repeated exposure to toys responsive to touch, we provided infants with 14 days of repeated reaching exposure with toys that moved and sounded *only* upon hand-toy contact. Working with the assumption that hand-toy contact providing haptic feedback is already a valuable movement consequence for the selection of appropriate reaching responses, we predicted that using contingently activated toys would further enhance the consequence of hand-toy contact and aid the creation of an even stronger reaching-specific value function over time. The discovery of the contingency between movement and consequence would drive infants’ persistence to repeat such an event, and thus, enhance and sustain the action-perception cycle even more. This would lead to a significant increase in reaches over time, where the target is being visually attended relative to hand-toy contacts happening without visually attending the target. In addition, we would expect to see a change in movement patterns, as revealed by kinematic measures that are appropriate to the reaching context.

Second, work on infant attention suggests that we could also enhance infants’ initial selective process by increasing infant object-directed attention. Specifically, empirical work guided by the intersensory redundancy hypothesis revealed that if an event’s sounds and motions are synchronous in a visual scene, infants will attend and perceptually process that event more than any other elements in the scene (Bahrick and Lickliter, 2000; Bahrick et al., 2004; Reynolds et al., 2014). Thus, in the context of early reaching, exposure to autonomously activated, synchronous moving and sounding toys in the reaching space may increase infants’ toy-directed attention. A by-product of such toy-directed attention may be greater attempts at toy-directed reaching activity, which could increase the likelihood of hand-toy contact and, subsequently, enhance infants’ persistence at reaching for the toy. This persistence may ultimately aid the discovery and selection process. But, in this case, the task differed from the condition described above in the key point that toy motion is *independent* from hand-toy contact, and therefore not a direct consequence of contacting the toy. To examine the developmental trajectory of reaching as a function of autonomously activated, synchronous moving and sounding toys, we provided infants with 14 days of repeated exposure to such self-activated toys. We inferred that if the moving and sounding toy captured infants’ attention, then infants would look at the toy more and show higher amounts of movement activity to attempt to reach for the toy. This, in turn, could increase the likelihood of hand-toy contact, thereby creating a reaching-specific value function possibly aiding the selection of successful movements for the reaching task. Also, with the increased reaching attempts we would expect a concomitant increase in movement kinematics appropriate to the reaching context over time.

## Materials and Methods

### Participants

Thirty-five infants, recruited within the week prior to turning 3 months of age participated in this study. Twenty-two were randomly assigned to one of two conditions: (a) Contingent (*n* = 11; six females, five males): the toy motion and sound was contingent on hand-toy contact, and (b) Continuous (*n* = 11; six females, five males): the toy motion was independent from hand-toy contact. A Control group (*n* = 13; six females, seven males) was from Williams et al. (2015b): in this group the toy did not move or sound. Based on parental reports, all infants included in the final sample were born full term and possessed no known sensory, motor, or neurological impairments. Also, no infant demonstrated the ability to successfully reach for and contact toys on the first day of the study. We followed the 22 infants in our two intervention groups for 16 consecutive days (1 day pre-test, 14 days intervention, 1 day post-test). The 13 control infants were only seen on the first pre-test day and last post-test day, which corresponded to day 16 in the intervention groups. This study and all procedures were approved by the Institutional Review Board of the University of Tennessee. Parents received an explanation of the study procedures and were shown the laboratory and equipment to be used prior to consenting participation. They were informed that their participation was voluntary and that they could withdraw their child from the study at any time without penalty. Parents received $5 on day 1 and on day 16 and a baby book containing a collection of pictures capturing the daily progresses of their infant’s reaching.

### Materials

#### Infant Seat and Table

**FIGURE 1:**
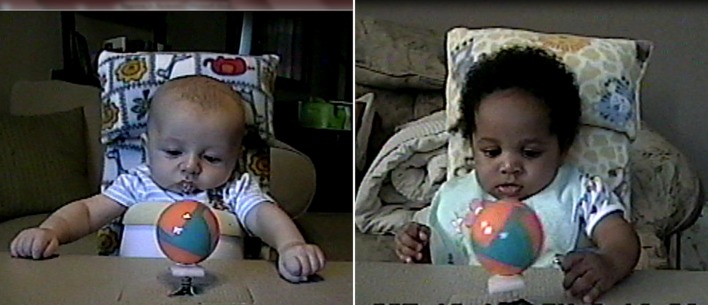
**Screenshot of gaze camera to depict the experimental setup for contingent **(Left)** and continuous **(Right)** conditions**.

During all testing sessions infants sat in a custom-designed infant seat reclined 10 degrees from vertical. A foam strap around infants’ torsos provided full postural support and permitted a full range of motion of the limbs. We placed the seat directly behind a wooden table (15′′ wide × 25′′ long × 15′′ high) which we used for toy presentation. The table height was waist high for all infants (see **Figure 1**).

#### Laboratory Toys

Toys used in the laboratory sessions were a mixture of small, colorful plastic spheres (5 cm diameter) comprised of non-toxic materials. These objects did not move nor make sound even upon hand-toy contact. Toys used for laboratory sessions of the control infants were a mixture of small, visually attractive colorful Peek-a-Blocks and plastic animal squirt toys (5–6 cm in diameter; see Williams et al., 2015b). These toys were used with the control group infants in place of the colorful plastic spheres to maintain their attention and motivation to the task given that infants in this group were never exposed to sounding and moving toys.

#### Home Toys

Toys for the contingent group moved and sounded only upon hand-toy contact. We modified the small, colorful plastic spheres by placing a bell inside. For trials, we placed each toy atop a small, Velcro-covered platform on a 1 ½ inch stiff spring. The spring securely sat on top of a 3 ½ inches tall × 4 ½ inches wide wooden block which fit snuggly through a hole on the surface of the custom-made wooden table cover. Once the block was inserted in the table hole, its top was flush with the top of the table so that only the toy on the spring extended through the table. A plain uniformly colored cover atop the table provided a smooth surface around the toy. Toys mounted on the spring for the contingent group oscillated and sounded with the smallest of hand-toy contact.

Toys for the continuous group were the same small colorful plastic spheres as for the contingent group. However, they sat on top of a Velcro-covered platform on a 1 ½ inch stiff plastic rod. The rod rested securely into a 3 ½ inches tall × 4 ½ inches wide custom-designed toy motor. All parts of the toy motor were encased in a hard plastic covering which also fit snuggly in the table surface opening so that the top of the motor casing was flush with the table cover. A button located under the table on the side of the motor casing activated the toy such that the toy oscillated with the sounding bell inside in a left-right motion for one full minute and then autonomously shut off. Thus, toy oscillations were independent of hand-toy contact.

#### Behavioral Recording

For laboratory pre- and post-tests, three video cameras captured the looking and reaching behavior of the infants. One camera, placed directly across the table at infant eye level captured gaze and reaching behavior while the two remaining cameras, situated 90 degrees left and right of the infant, captured the movements of each arm. A digital video switcher (Datavideo Corp., Whittier, CA, USA) merged the images from the two lateral cameras to create a split-screen image and sent it to a VCR for recording. For home sessions, only the video camera situated directly across the table at infant eye level was used. It captured both gaze and reaching behavior of the infants and recorded it to a VHS-C cassette.

During all laboratory sessions a Mini Flock of Birds motion analysis system (Ascension Technology Corp., Burlington, VT, USA) captured arm kinematics. The experimenter applied a mini bird marker (8 mm) to the dorsal side of each wrist and secured the wires up the arm and behind the infant seat with hypoallergenic tape. The Mini Flock of birds sampled movement at 120 Hz. We synchronized the video and kinematic recordings with a frame counter (Horita, Mission Viejo, CA, USA) superimposed on the video recording that started and stopped when the experimenter started and stopped the motion analysis system.

### Procedure

Testing occurred in three phases: (a) Pre-intervention assessment (day 1 in laboratory), (b) 14-day sensory-motor intervention (days 2–15 in home), and (c) Post-intervention assessment (day 16 in laboratory). The contingent and continuous groups participated in all three phases of the study while the control group only participated in the pre- and post-assessment phases.

#### Laboratory Pre-intervention Assessment

During this phase, we established baseline measures of gaze and reaching. This phase was identical for all three groups of infants. The experimenter secured the infant in the seat and placed the seat behind the table. Prior to applying the Mini Flock of Bird markers, the experimenter collected one kinematic trial with one marker on the table top to denote the toy location for all trials. Trials began with one experimenter seated across the wooden table from the infant with a toy in hand. After capturing the infant’s attention, the experimenter placed one toy on the pre-determined toy position at midline and 14 cm from the edge of the table where the infant sat (Williams et al., 2015b). Toys for this phase were all non-moving and silent. A second experimenter triggered the motion analysis system and tracked trial duration. Once the toy was on the table top, the experimenter situated across from the infant remained silent and did not interfere with infant behavior in the presence of the toy. We collected 10, 1-min trials. During each trial, all infants had the opportunity to repeatedly reach for and contact the toys. Only infants who performed zero hand-toy contacts during this initial phase of the study were entered in the study and continued to the next phase as done in Williams et al. (2015b).

#### Home Sensory-motor Intervention

Two experimenters traveled to the infants’ homes for those assigned to the contingent and continuous groups to provide the 14 daily sensory-motor experience sessions. Home sessions occurred in a low distraction area of the home and in a similar manner as the pre-intervention assessment session. After securing the infant in the seat, one experimenter sat directly across the table from the infant, captured the infant’s attention, and placed one object at midline and 14 cm in front of the infant. For the continuous group, the experimenter switched on the motor immediately after placing the toy on the table. Again, once the toy was in place, the experimenter remained silent and did not interfere with the infant’s behavior while the second experimenter kept track of trial duration. As in the pre-intervention assessment session, we collected 10, 1-min trials. Again, on each trial, infants had the opportunity to repeatedly reach for and contact the toys.

#### Laboratory Post-intervention Assessment

During this phase, we reassessed all infants’ gaze and reaching behaviors after the 14-day sensory-motor intervention, or no intervention (control). We conducted this session in exactly the same fashion as the pre-intervention assessment on the first day of the study.

### Data Coding and Analysis

We coded all video recordings of gaze and reaching behavior with The Observer XT-9 (Noldus Information Technology, Wageningen, The Netherlands). All kinematic data were processed with a custom-made MATLAB program (The Mathworks, Inc., Natick, MA, USA). We conducted all analyses on the 1-min time periods when toys were in infants’ reaching spaces. Also, with regard to statistical analyses, we used parametric analyses when data met all appropriate test assumptions. Otherwise, we used non-parametric analyses.

#### Reaching Measures

Two independent coders scored the number of hand-toy contacts (visually attended and non-visually attended). These coders overlapped on 20% of the sample in order to compute interobserver reliability, which reached a 91% agreement or above for each infant. As in Williams et al. (2015b), we coded a visually attended contact when the infant looked at the toy prior to, during the arm movement toward the toy, up until hand-toy contact. If the infant shifted their gaze away from the toy during this time reaching window we considered the hand-toy contact non-visually attended. Key computations included the total number of non-visually attended and visually attended target contacts, as well as a visually attended target contact index. In a similar way that Hinojosa et al. (2003) calculated handedness, we calculated the visually attended target contact index (a *z*-score), to capture the relative distribution in amounts of visually attended and non-visually attended target contacts in a single measure. Specifically, we calculated a difference score between the number of visually attended target contacts and the number of non-visually attended target contacts, then divided the difference by the square root of the sum of contacts. These standardized scores gave us clear benchmarks for comparison on amount of visually attended target reaching responses.

#### Looking Measures

The videos from the front camera capturing the infant gaze were coded in the Observer XT (Noldus, Inc.) by two trained independent coders who scored the onsets and offsets if the infant looking behavior according to five looking areas: Toy, experimenter, right hand, left hand, or elsewhere. Elsewhere was coded when infants looked anywhere other than the four areas (i.e., look at the table) or when we could not determine gaze location. Coders overlapped on 20% of the sample and interobserver reliability reached an agreement of 85% or above for each infant. Such coding of looking measures from video recordings along with interobserver reliability is a standard procedure in infant studies (e.g., von Hofsten, 1982, 1984; Ruff and Rothbart, 1996; Clearfield and Mix, 1999; for a comprehensive review of visual attention measures). Looking measures are reported herein as the percent of trial duration spent looking to each area.

#### Kinematic Measures

A custom-made MATLAB program filtered the movement time series with a zero-phase, second-order Butterworth filter with a 6 Hz cut-off and transformed the time series into 3-D resultant hand-toy distance and velocity profiles for each hand. We focused our kinematic analyses on the preferred reaching hand during times when infants looked at the toy. We defined the preferred reaching hand as the hand used most frequently by infants in the post-intervention assessment phase of the study. If infants in the contingent and continuous groups did not perform enough contacts during that phase to use this criterion, we selected the hand that infants used most frequently during the sensory-motor intervention phase of the study. If infants in the control group did not perform enough contacts in the post-intervention assessment phase then we selected the hand with the lowest movement velocity during the reaching task as the preferred reaching hand. We used this velocity-based criterion as prior research indicates that as infants approach the emergence of reaching, velocity during reaching tasks tends to decline (Bhat et al., 2005).

To analyze toy-directed behavior, we analyzed the kinematic times series associated with time periods during which infants looked at the toy. To determine the portions of the time series corresponding to when infants looked at the toy, we synchronized the lateral reaching cameras, which contained the time-frame counter for the kinematics, with the gaze camera. Once synchronized, we recorded the kinematic time codes corresponding to periods when infants looked at the toy and entered these into the MATLAB program.

Kinematic computations included the mean time that the preferred reaching hand spent within 10 cm of the toy (Williams et al., 2015b), which we computed based on the resultant distance between the preferred hand position and the pre-determined toy position. Also, we computed the mean peak velocity of the preferred hand. The MATLAB program analyzed the velocity profile with a 3-point technique in order to determine peaks in the profile. Once the program identified the peaks, it divided the sum of all peak values by the total number of velocity peaks identified to produce a mean peak value.

Control group values for pre- and post-test days are shown on each graph.

## Results

### Exposure Time

Due to sporadic fussiness not all infants in the contingent and continuous conditions completed 10 full trials each day. However, overall task exposure times, in total minutes, did not differ significantly between the contingent (*M* = 103.45, *SEM* = 2.56) and continuous [*M* = 107.55, *SEM* = 13.74, *t*(20) = 0.840, *p* (2-tailed) = 0.411, *d* = 0.36] groups.

### Reaching

#### Total Contacts (**Figure 2**)

Separate Wilcoxon Signed-Ranks tests revealed that infants in the contingent (*M*_Day16_ = 27.64, *SEM*_Day16_ = 11.17), continuous (*M*_Day16_ = 7.91, *SEM*_Day16_ = 2.81), and control (*M*_Day16_ = 6.46, *SEM*_Day16_ = 4.47) groups exhibited significant increases in total contacts between pre- and post-intervention day [*Z* = -2.675, *p* (2-tailed) = 0.007, *r* = 0.81, *Z* = -2.521, *p* (2-tailed) = 0.012, *r* = 0.76, *Z* = -2.032, *p* (2-tailed) = 0.042, *r* = 0.56, respectively]. Further, curve estimation analyses over the 16-day period showed that the contingent and continuous groups displayed significant linear growth in total contacts [*F*(1,14) = 65.514, *p* < 0.0001, *R*^2^ = 0.82, *F*(1,14) = 16.918, *p* = 0.001 *R*^2^ = 0.55, respectively]. However, planned Mann–Whitney comparisons with the control group on post-intervention day values revealed that the continuous group did not display significantly more contacts relative to the control group [*U* = 46.50, *Z* = -1.524, *p* (2-tailed) = 0.128, *r* = 0.31], while the contingent group displayed significantly higher total contacts relative to the control group [*U* = 38.00, *Z* = -2.017, *p* (2-tailed) = 0.044, *r* = 0.41].

#### Visually Attended Target Contact Index (**Figure 3**)

The index of visually attended target contacts (*z*-score) provides (a) a single measure that captures the relative amount of visually attended and non-visually attended hand-target contacts performed over time and (b) a measure that allowed clear benchmarks for comparisons between the groups. **Figure 3** reveals that infants in the contingent and continuous groups displayed similar ratios of visually attended/non-visually attended target contacts during the first week of intervention but, from Day 8, the two groups began to diverge. Infants in the contingent group increased their number of visually attended hand-toy contacts relative to non-visually attended contacts as the study progressed, while the continuous group did not. **Figure 3** also shows that on all but 2 days after Day 8, infants in the contingent group had a visually attended target contact index that was greater than 1 standard deviation unit relative to the continuous group index, and on four of those days, the contingent group *z*-score values were above 1.65 (90% confidence level).

#### High versus Low Performers (**Figure 4**)

To gain more insights into these data, we examined whether the number of visually attended target contacts performed by the infants on the post-intervention day was an accurate reflection of the hand-toy contact history performed during the prior intervention days. This was relevant because there was much between subject variability on the last day performance and toy motion and sound were removed on post-test day. All of our three samples contained infants who did not produce any visually attended target hand-toy contacts on that last day despite the 14-day intervention (36% in the contingent group and 27% in the continuous group, compared to 69% in the control group). Some other infants produced as little as 1, 2, or 3 visually attended target contacts on the last day, and some contacted the toy quite often. Here we asked whether the infants with higher contact performance on the last day, were also the infants who most likely discovered the consequences of their actions through their contact history. Likewise, we asked whether the poor performers on the last day of the study were also the ones with a history of lower visually attended target contacts over the 14-day intervention. We anticipated that this analysis would shed further light on the respective impact our interventions on the process of discovering action consequences on learning to reach.

To examine this question, we used the group medians to split infants into high performers (those displaying hand-toy contacts above the group median) and low performers (those at and below the group median) based on the number of visually attended target contacts performed on the last day of the study. Then, we examined whether the last day performances were in line with the observed 14-day intervention progression. **Figure 4** presents the high versus low performers in the contingent group on the left panel and the high versus low performers in the continuous group in the right panel. A 2 (Performance Group) × 16 (Day)repeated measures ANOVA on the contingent group using a Greenhouse–Geisser adjustment for lack of sphericity revealed a significant main effect of Performance Group [*F*(1,9) = 13.492, *p* = 0.005, η^2^ = 0.600], Day [*F*(2.691,24.222) = 4.713, *p* = 0.012, η^2^ = 0.344], and Performance Group × Day interaction [*F*(2.691,24.222) = 4.817, *p* = 0.011, η^2^ = 0.349]. The same analysis performed on the continuous group revealed no significant differences between Performance Groups, nor Days (all *p*-values > 0.154). This indicated that high versus low performance groups only differed in the contingent group. A last analysis, aimed at comparing high performers between intervention groups, revealed a main effect of Group [*F*(1,8) = 7.429, *p* = 0.026, η^2^ = 0.481], and Day [*F*(3.213,25.701) = 4.566, *p* = 0.010, η^2^ = 0.363], but no Group × Day interaction [*F*(3.213,25.701) = 2.691, *p* = 0.064, η^2^ = 0.252]. Thus, in conclusion, when we split infants based on the number of visually attended target contacts performed on the last day, we were able to show: (a) that the last-day performance accurately captured the history of contacts performed throughout the intervention period, and (b) that only the high-performing infants in the contingent group benefitted from the contact enhancement intervention by displaying a growing history of hand-toy contacts. No infants in the other performing groups did.

### Looking

#### Day 1

To assess whether our intervention groups differed in their distribution of looking behavior at the start of the study, we performed a 3 (Group) × 5 (Look Area) repeated measures ANOVA on the Day 1 normalized looking durations. To adjust for a violation of sphericity we applied a Greenhouse–Geisser correction. There was no Group effect [*F*(2,32) = 0.839, *p* = 0.441, η^2^ = 0.05] and no Group by Look Area interaction [*F*(2.906,46.504) = 2.727, *p* = 0.056, η^2^ = 0.146]. However, there was a significant effect of Look Area [*F*(1.453,46.504) = 59.370, *p* < 0.0001, η^2^ = 0.65]. Overall, infants looked at the toy the most (52.55%), then elsewhere (32.48%), then at the experimenter (12.73%), then at their left hand (2.52%), and least at their right hand (0.284%). All pairwise comparisons between the looking areas were significant at the α = 0.05 level.

#### Day 16

To assess whether our intervention groups differed in their distribution of looking behavior at the end of the study, we performed a 3 (Group) × 5 (Look Area) repeated measures ANOVA on the Day 16 normalized looking durations. Again, we used a Greenhouse–Geisser adjustment for a sphericity violation. There was no Group effect [*F*(2,32) = 0.550, *p* = 0.582, η^2^ = 0.033]. As on Day 1 we found a significant effect of Look Area [*F*(1.420,45.427) = 131.895, *p* < 0.0001, η^2^ = 0.805]. Overall, infants looked at the toy the most (48.71%), then elsewhere (39.78%), then at the experimenter (8.59%), then at their left hand (1.82%), and least at their right hand (1.45%). Pairwise comparisons revealed that in the Contingent and Continuous groups, infant looked significantly more at the toy than all other look areas (*p* < 0.05) but showed equal looking elsewhere. The control group looked significantly more at the toy than all other look areas, including elsewhere (*p* < 0.05). Further, there was a significant Group by Look Area interaction [*F*(2.839,45.427) = 4.626, *p* = 0.007, η^2^ = 0.224]. Within toy, experimenter, and left hand look areas, groups did not differ. Control infants looked significantly more at their right hand relative to continuous infants (*p* = 0.042). Also, control infants spent significantly less time than contingent infants (*p* = 0.001) and marginally less time than continuous infants (*p* = 0.065) looking elsewhere.

#### Over the Study Period

**Figure 5** depicts the percent of trial duration that infants in the contingent and continuous groups looked to each area from pre- to post-intervention. To examine whether the distribution of looking behavior changed over time, we performed a 2(Group) × 5 (Look Area) × 16 (Day) repeated measures ANOVA, with a Greenhouse–Geisser correction. There were no main effects of Group [*F*(1,20) = 1.000, *p* = 0.329, η^2^ = 0.048] nor Day [*F*(1,20) = 1.885, *p* = 0.329, η^2^ = 0.048], but a main effect of Look Area [(1.153,80) = 75.025, *p* < 0.0001, η^2^ = 0.790]. Overall, infants tended to look elsewhere the most (45.18%), then at the toy (44.49%), then at the experimenter (8.73%), then at their left hand (1.13%), and least at their right hand (0.42%). There was also a Look Area by Day interaction [*F*(9.038, 20) = 3.945, *p* < 0.0001, η^2^ = 0.165] indicating a change in looking behavior over time in some Look Areas, but not all. Follow-up testing confirmed that in both intervention groups, infants decreased their amount of looking at the toy over time, while they increased their amount of looking elsewhere [*F*(7.245,20) = 4.866, *p* < 0.0001, η^2^ = 0.196]. These trends in looking behavior did not differ between high and low performers in either intervention groups.

### Kinematics

**Figure 6** (left) reports the mean peak velocity of the preferred reaching hand on the pre- and post-test days of the study for all three groups. A 3 (Group) × 2 (Day) repeated measures ANOVA revealed no significant effect of Group [*F*(2,32) = 0.668, *p* = 0.520, η^2^ = 0.04] nor of day [*F*(1,32) = 0.314, *p* = 0.579, η^2^ = 0.01] but, there was a significant Group by Day interaction [*F*(2,32) = 4.785, *p* = 0.015, η^2^ = 0.23]. *Post hoc* analyses indicated that while the contingent and control groups showed no significant change in peak velocity, the continuous group displayed a significant increase in peak velocity between pre- and post-test day (*p* = 0.011).

**Figure 6** (right) displays the mean percent of time infants had the preferred reaching hand within 10 cm of the toy between pre- and post-test days of the study. Separate Wilcoxon Signed-Ranks tests revealed that only the contingent group exhibited a significant increase in the time spent within 10 cm of the toy [*Z* = -2.490, *p* (2-tailed) = 0.013, *r* = 0.75]. The increases displayed in the continuous and control groups were not significant [*Z* = -1.156, *p* (2-tailed) = 0.248, *r* = 0.35, *Z* = -1.642, *p* (2-tailed) = 0.101, *r* = 0.46, respectively].

## Discussion

In this study, we examined the impact that hand-toy contact consequences had on the developmental trajectories of infant reaching behavior. Specifically, we manipulated the context in which toy sound and motion would be activated to examine how such context enhancements could augment infants’ persistence at reaching for the toys. A critical difference between intervention groups was that while one group (contingent) experienced such enhancement solely during successful hand-toy contacts, the other group (continuous) was able to experience such enhancement continuously whether attempting to reach or not and independently from successful hand-toy contacts. Using tenets drawn from Dynamic Systems Theory, the TNGS, and Approximate Optimal Control, on the one hand, and the Intersensory Redundancy Hypothesis on the other, we predicted that if the toy manipulations served to enhance the action-perception cycle, then infants in both intervention groups would increase the frequency of hand-toy contact with the moving and sounding toy, compared to the non-intervention control group which had visually attractive, yet still and silent toys. Such findings would support the interpretation that those infants, in each intervention group, followed different routes to discover and select effective arm movements for the reaching task. But, our results revealed that infants in the contingent group were the ones who benefitted the most from their intervention.

**FIGURE 2:**
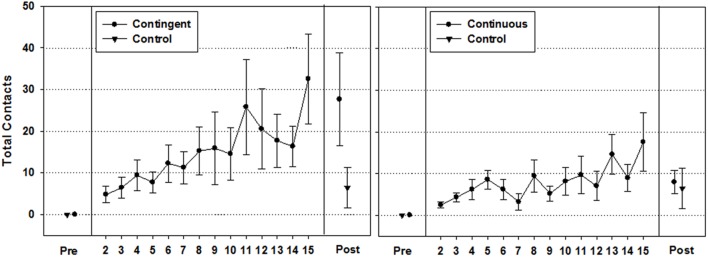
**Mean total contacts (±1 *SEM*) for the contingent **(Left)** and continuous **(Right)** groups by day**.

**FIGURE 3:**
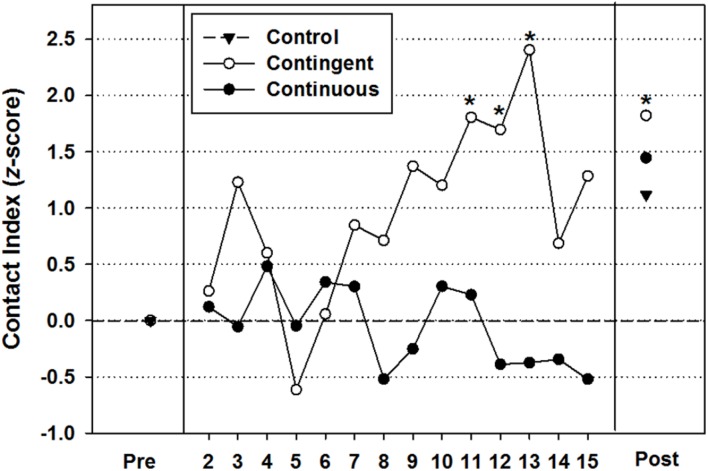
**Mean visually attended contact indices (*z*-scores) for each group by day.** The dashed line at zero indicates no preference for visually attended or non-visually attended contacts. Points with an asterisk are index scores that are greater than *z* > 1.65, indicating performance level with a 90% confidence.

**FIGURE 4:**
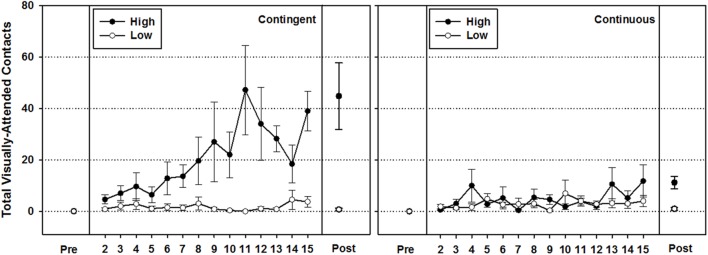
**Mean total visually attended contacts (±1 *SEM*) for high and low performers in the contingent **(Left)** and continuous **(Right)** groups**.

With regard to the amount of total hand-toy contacts, both intervention groups showed significant gains in reaching from the first to the last day of the study, but only the contingent group produced hand-toy contact amounts that were significantly greater than those produced by the control group (**Figure 2**). Furthermore, the visually attended target contact index indicated that infants in the contingent group began to diverge from the continuous group about half way through the study by performing more visually attended target contacts relative to non-visually attended target contacts (**Figure 3**). To gain a better sense of the immediate impact that the varied experiences available to the contingent and continuous conditions had on the development of reaching behavior, we focused in on the variability in infants’ performances on the post-intervention day and traced it back to the history of hand-toy contact observed during the intervention. When doing so, results revealed that only higher performing infants in the contingent group showed significant gains in contacts over time while infants in the continuous group did not (**Figure 4**).

Generally speaking, the observed increase in visually attended target contacts is consistent with prior research in that repeated opportunities to actively attempt reaching behaviors and perceive the behavioral consequences may be enough to drive the reaching selection process (Bojczyk and Corbetta, 2004; Lobo et al., 2004; Williams et al., 2015b). Theoretically, infants in both intervention groups were able to explore the reaching task, perform various reaching movements, experience direct hand-toy contact, and gradually select those movements that met task demands based on the developing value function. However, interestingly, our intervention groups indicated that only infants in the contingent condition, where toy motion and sound occurred only in response to their successful action, made significant progress over time. Those infants presumably discovered the association between making contact with the toy and eliciting toy motion and sound as a direct consequence of their movement. In the continuous group, infants could experience toy motion and sound, but it was independent of their action. Consistent with the above mentioned theoretical frameworks, interactions with a responsive toy contributed to highlight a successful reaching movement, which, in turn, spurred the action-perception cycle, permitting further refinement of the developing value function and allowing infants to more effectively select reaching movements that met the immediate task demands (Jennings et al., 1979; Edelman, 1987; Thelen and Smith, 1998; Berthier et al., 2005; Williams et al., 2015a). Further support may be seen in the visually attended target contact index analyses where infants in the contingent group began to display many more visually attended to non-visually attended target contacts over time relative to the continuous group. Based on these results, we believe that the contingent infants were more effectively, and efficiently, selecting the successful reaching movements over time through the creation, refinement, and use of a specific value function.

**FIGURE 5:**
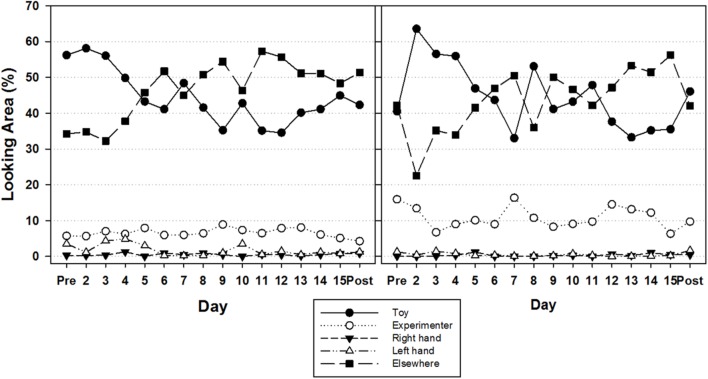
**Mean percent trial of looking duration by Look Area for infants in the contingent **(Left)** and continuous **(Right)** groups**.

The looking analyses revealed no differences between intervention groups with regard to visual attention allocation. At the start of the study, all three groups demonstrated equal distributions of looking patterns toward the different looking categories (toy, experimenter, right hand, left hand, elsewhere). All infants spent significantly more time looking at the toy and elsewhere relative to the other categories. Over the course of the 16-day study, however, visual attention to the toy declined despite remaining overall relatively high compared to the other looking categories. Conversely, the direction of visual attention to elsewhere increased over time (**Figure 5**). This change in visual attention allocation did not affect the rate of toy contact, since it continued to increase over time (see Williams et al., 2015b for similar findings). More surprising, however, was the fact that we did not find differences in looking behavior between intervention groups. We designed the toy for the continuous group based on work in the area of intersensory redundancy (Bahrick and Lickliter, 2000; Bahrick et al., 2004). As stated in the introduction, we expected the autonomously activated, moving, and sounding toy to capture infants’ visual attention more and subsequently drive higher amounts of persistence in trying to touch the moving and sounding toy. Our looking analyses clearly revealed that this did not occur. A possible explanation for these results is that looking time and level of attention are distinctly different. For instance, psychophysiological work has shown that infants shift their level of information processing during single looks toward stimuli. Through measures of heart rate variability, infants shift from stimulus orienting, to sustained attention (active information processing), and attention termination all within the same look to a stimulus (Richards, 1997; Reynolds and Richards, 2008). It is possible that the monotony of the toy motion did not serve to attract visual attention and maintain sustained attention to the toy as much as we thought it would in this group. Another possibility is that the low amount of hand-toy contacts in the continuous group compared to looking times that are equivalent to those of the contingent group, reflect a different attention-action ratio than the one present in the contingent group. We can speculate that infants in the continuous group may have spent more time processing the synchronous, multimodal event in an attempt to map those perceptual characteristics to their movements to meet task demands. The kinematic results, which we discuss below, may provide an indirect assessment of such an ongoing process.

**FIGURE 6:**
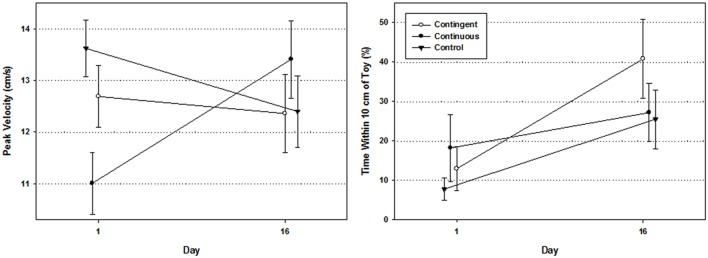
**(Left)** Mean peak velocity of the preferred reaching hand by group. **(Right)** Mean percent of time that preferred reaching hand was within 10 cm of the toy.

The kinematic analyses on the first and final days of the study revealed that infants in the continuous group modulated their arm movements presumably to match immediate task demands, while infants in the contingent and control group did not. Specifically, infants in the continuous groups displayed a significant increase in peak speed over the course of the study (**Figure 6**, left). These results may indicate that these young infants capitalized on their respective sensory-motor experiences to select different kinds of movements with particular motor control characteristics to match the varying task demands. In the typical reaching situation, with stationary toys, lower peak movement speeds are associated with better reaching control while higher peak speeds typically indicate less control (Thelen et al., 1993, 1996; Bhat et al., 2005). However, the infants in the continuous group may have learned, through their particular sensory-motor experience, that if they selected more rapid reaching movements, they would increase their chances of contacting the moving target. von Hofsten and Lindhagen (1979) showed that infants at the initial transition to reaching for stationary objects are also capable of reaching for moving ones. We can infer from their results that infants modified their reaching speeds to accomplish their task but in our study we explicitly showed the kinematic changes. Theoretically, infants’ value functions built up through their respective sensory-motor experiences in such a way as to drive the selection of movements, even at the kinematic level, to meet task demands.

As hinted above, the peak speed results for infants in the continuous group may also potentially be related to their visual attention. It is possible that the synchronous, multimodal event (toy motion and sound) truly drove their attention and allowed them to perceive and extract specific characteristics of the multimodal event such as rate and rhythm. Specifically, the continuous infants may have spent more time sustaining their attention to process the characteristics of such a multimodal event (Bahrick and Lickliter, 2000; Bahrick et al., 2004; Reynolds and Richards, 2008). Consequently, those infants may have also been slower at mapping their motor behavior onto the moving toy pattern to make contact and one strategy available for success would be to increase movement speed.

Also, our kinematic analysis showed that the contingent group was the only group to show a significant increase in the amount of time spent with the preferred reaching hand near the toy. We know that infants gradually move their arms closer to midline (White et al., 1964; von Hofsten, 1984; Spencer and Thelen, 2000) and this is true of our results as well. All infants did show an increase in the amount of time spent with the preferred reaching hand near the toy. However, only the contingent group’s increase reached statistical significance. Again, we believe that over the course of the study, having been exposed to the contingently activated link between reaching movements and successful outcome, the contingent infants developed a more precise value function which allowed them to select movements that would increase the likelihood of success in the reaching situation.

A potential limitation of this study may be a focus only on short-term consequences of the sensory-motor intervention. Indeed, many of the more recent investigations into the impact of early sensory-motor interventions have focused on reaching movements in 6-month-old or younger infants (Needham et al., 2002; Lobo et al., 2004; Lobo and Galloway, 2008, 2013; Libertus and Needham, 2010; Lee and Newell, 2013; Williams et al., 2015b). Based on prior research, we know that various types of early sensory-motor experiences have immediate short-term consequences on early infant reaching and exploratory behavior. However, with the prediction made by many studies that the emergence of reaching has an impact on all domains of development (Fogel et al., 1992; Bushnell and Boudreau, 1993; Fogel, 1997; Thelen and Smith, 1998; Eppler, 1995; Corbetta and Snapp-Childs, 2009) it is important for future research to go beyond just investigating the short-term consequences of early sensory-motor experience. Rather, after examining the emergent developmental trajectories of reaching behavior as a function of early sensory-motor experience, researchers should examine how such experiences could lead to distinct cascadic effects over developmental time. As reviewed in the introduction, reach onset entails a number of behavioral ramifications at multiple levels. Understanding how these ramifications could be tied to specific early perpetual-motor experiences is an important developmental question.

Another potential limitation may be the fact that the control group on days 1 and 16 was presented with different toys than the infants in the contingent and continuous conditions. Thus, it could be possible that the observed differences in reaching behavior between our intervention conditions and the control group resulted from using different toys. These toy differences could have captured infants’ attention differentially, and in turn, affected the amount of reaching behavior produced, particularly on day 16. While it is true that we discovered differences in reaching behavior, we did not find such differences to be related to differences in looking behavior. Our analysis of day 16 looking behavior revealed that infants in the contingent, continuous, and control groups spent equal amounts of time visually attending the toys. Also, the control infants were the only group to allocate significantly more time looking to the toy than the other four look areas, including elsewhere. If infants in the control group had lacked interest in the toys, we would have seen less looking at the toy relative to both the other conditions and look areas. Thus, despite these between group toy differences, it does not appear that looking results on the final day may have driven the observed differences in reaching behavior

Finally, another limitation of this study may concern the lack of a continuous control group receiving daily exposure with toys that are not sounding nor moving. Such a control group could have provided a better baseline to estimate the added impact of our contingent and continuous intervention conditions on reaching development. The reason we did not collect such a basic repeated task exposure group as part of this study is because we already had tested a group similar to that condition in our prior study (Williams et al., 2015b). If we compare results from that prior study with results from the present study, we find that the contingent group displayed the steepest reaching progress over time, followed next by the repeated exposure group (Williams et al., 2015b), and finally followed by the continuous group with the less steep progress.

We contend that our results are in agreement with tenets of the Dynamic Systems Theory, TNGS, and Approximate Optimal Control perspectives. However, a potential alternative theoretical account, especially for the results presented here, is straightforward operant conditioning. Indeed, the notion of contingency as it impacts the control and selection of behavior is central to that perspective (Skinner, 1974, 1981). However, a growing amount of research in the fields of neuroscience, perceptual, motor, emotional, and cognitive development, among others, indicates that even seemingly simple behaviors, such as that of reaching for and contacting a toy, is actually quite complex (Smith, 2005; Winkielman et al., 2015). Specifically with regard to reaching, contemporary research indicates that many subsystems contribute cooperatively to the performance of such a behavior (Thelen and Smith, 1994, 1998; Spencer et al., 2000; Clearfield and Thelen, 2001; Corbetta, 2009; Cunha et al., 2015). Among the factors underlying the learning of such a behavior, we do not discount the contribution of operant learning principles. On the contrary, such principles are at play in the learning-to-reach process, as evidenced by the centrality of reinforcement learning mechanisms aiding the development of the value function that aids the selection of reaching movements in Approximate Optimal Control perspectives (Barto, 2002; Berthier et al., 2005). However, an explanation based on operant conditioning alone falls short, in our opinion, in capturing all the factors and behavioral complexity tied to the emergence and subsequent development of new skills. In that sense, we see the combination of Dynamic Systems Theory, TNGS, and Approximate Optimal Control as complementing one another in accounting how brain, motor, perception, and experience all contribute to different extents to our understanding of the behavioral learning process observed.

In closing, contemporary theoretical perspectives such as Dynamic Systems Theory, TNGS, and Approximate Optimal Control argue that reaching behavior emerges gradually through repeated self-generated activity during the reaching task. Repeated exposure to the reaching situation offers infants opportunities to engage in continuous action-perception cycles during which they discover the consequences of various reaching movements, create and develop a value function from perception of such consequences, and subsequently use the value function to select those reaching movements that lead to the more positive outcomes (Edelman, 1987; Thelen and Smith, 1998; Berthier et al., 2005; Williams et al., 2015a). The notion that repeated task exposure, without external guidance, is enough to drive the emergence of reaching behavior has support across multiple types of reaching situations (Bojczyk and Corbetta, 2004; Lobo et al., 2004; Lobo and Galloway, 2008; Williams et al., 2015b). Furthermore, based on Schlesinger and Parisi’s (2001) work, it appears that early in the reaching process, the movement consequence of hand-toy contact and haptic feedback received, carries a high value and sparks the action-perception cycle to drive a more efficient selective process (Corbetta et al., 2015; Williams et al., 2015b). In this study, we capitalized on adding further consequences to the hand-toy contact event by providing infants with responsive toys, which moved and sounded only upon contact. Based on the results, we infer that the contingently activated toy highlighted the movement consequence of hand-toy contact, which increased the repetition of action-perception cycles. This repetition of action and perception drove the creation of a precise value function that allowed infants in the contingent condition to more efficiently discover and select reaching movements adaptive to the task-at-hand.

## Author Contributions

JW designed study, collected the data, did most of the analyses, and wrote the first draft of the manuscript. DC contributed to design of the study, helped complete data collection, performed additional analyses, and contributed to writing and revising of first draft provided by JW.

## Conflict of Interest Statement

The authors declare that the research was conducted in the absence of any commercial or financial relationships that could be construed as a potential conflict of interest.
